# Simplified Internal Audits of the Welfare Quality Protocol in Dairy Farms: Are They Effective in Improving Welfare Practices?

**DOI:** 10.3390/ani15020237

**Published:** 2025-01-16

**Authors:** Maria Francisca Ferreira, Catarina Stilwell, George Stilwell

**Affiliations:** 1 Concerta International Consulting, 1050-215 Lisbon, Portugal; franciscarcferreira@gmail.com (M.F.F.); catarinastilwell@concerta-international.com (C.S.); 2 Animal Behaviour and Welfare Research Laboratory, Centre for Interdisciplinary Research in Animal Health, Veterinary Medicine Faculty, Lisbon University, Av. Universidade Técnica, Alto da Ajuda, 1330-477 Lisbon, Portugal; 3 Associate Laboratory for Animal and Veterinary Sciences (AL4AnimalS), Faculty of Veterinary Medicine, University of Lisbon, 1300-477 Lisbon, Portugal

**Keywords:** welfare quality network, welfare, dairy cattle, certification, audits

## Abstract

The Welfair^®^ certificate has become an important part of food chain integrity for animal welfare assessment in several countries, relying on a rigorous audit that verifies compliance with legislation and assesses animal welfare through the Welfare Quality Protocol (WQP). Dairy cattle farmers are encouraged to conduct internal audits beforehand to self-assess the farm’s animal welfare level. Since early 2023, the Welfair^®^ scheme has proposed simplified audits to shorten the time needed for internal audits. Ten measures are selected from the WQP, five of which must always be assessed: body condition, water provision, lameness, integument alterations, and pain management in disbudding. The main objective of this study was to determine whether analyzing the results of these five key indicators helps in identifying welfare problems, ultimately leading to a better final score. To test this, seven Portuguese commercial dairy farms were randomly selected to conduct a simplified internal audit followed by a certification audit. Considering the circumstances of our study, the visits proved essential to promoting better welfare practices, which positively influenced the final classification. However, areas that require improvement (such as the lack of an accurate risk analysis of the simplified audits provided by the Welfair^®^ scheme) were identified and are discussed.

## Simple Summary

It is essential to regularly identify welfare issues and continuously improve welfare practices on dairy farms. Since early 2023, the Welfair^®^ scheme has provided simplified internal audits based on the Welfare Quality^®^ Protocols as a way to shorten the time and simplify the assessment process. We hypothesized that, even if a full assessment is not conducted, this practical approach is sufficient to drive changes in welfare conditions on farms, contributing to a better final classification. This study aimed to evaluate the feasibility and relevance of these visits, offering a critical analysis of the resulting improvements.

## 1. Introduction

In recent years, animal welfare has become a priority issue in many developed countries. Social concerns have spurred the development of science-based welfare assessment protocols to promote high standards of animal welfare throughout all stages of animal production. In Europe, to meet consumers’ expectations, the European Commission funded a project in 2009 to design methods for assessing the overall welfare of cattle, pigs, and poultry on farms and at slaughterhouses [1], known as the Welfare Quality^®^ Protocols (WQP).

**Figure 1:**
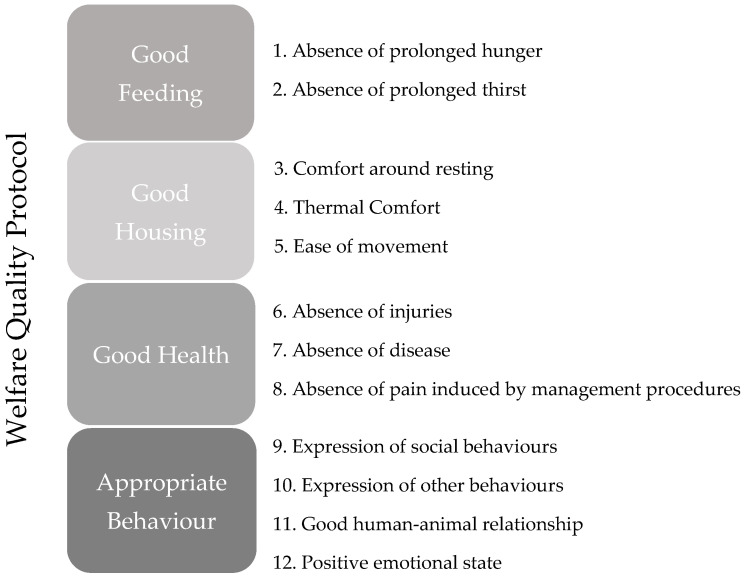
The principles and criteria that are the basis for the Welfare Quality Protocols [5].

These protocols primarily focus on animal-based indicators, with less emphasis on resources and management-based ones, aiming to reflect the physical, mental, and behavioral well-being of animals [2]. All protocols outline four principles: good feeding, good housing, good health, and appropriate behavior. For each principle, several animal welfare criteria are specified, as illustrated in Figure 1. Each criterion is evaluated through indicators that must meet three attributes: validity, feasibility, and reliability [3,4,5]. Consequently, indicators are tailored to specific production systems and species (for bovine, porcine, caprine, and ovine species, as well as for hens, chickens, rabbits, and turkeys).

A score is assigned to each criterion based on the assessment of their indicators. These criterion scores are then combined to calculate the respective principle score. The simple average of the four principles will result in the farm’s final classification, scaled from 0 to 100 points. Four classification levels are proposed: 0–19 (not acceptable), 20–54 (acceptable), 55–79 (enhanced), and 80–100 (excellent) [5].

This assessment can be routinely utilized by farmers and slaughterhouse managers to identify welfare issues and gradually improve welfare practices [1]. These standardized European assessments are the basis of the Welfair^®^ certification [6] for most farm species. This certificate aims to acknowledge the animal welfare status of farms, benefiting producers and slaughterhouse managers [6]. Any farm seeking the Welfair^®^ certification must undergo a rigorous animal welfare audit every year, which includes verifying compliance with European welfare legislation and applying the WQP. To achieve certification, a farm must attain a score of enhanced or excellent. This certification scheme is already playing a crucial role in ensuring food chain integrity by providing consumers with a differentiating factor when looking for a more animal-friendly production [6,7].

For the assessment of dairy farms, 30 indicators are proposed by the WQP for dairy cows. Of these, nine are resource-based (related to water provision and ease of movement) or animal management-based measures (including procedures for disbudding/dehorning, tail docking, access to outdoor loafing areas or pasture, and mortality rate) (Table 1). The Welfair^®^ scheme determines that prior to the certification audit, producers should conduct an internal audit to self-assess their animals’ welfare and address any deficiencies. To ensure validity and credibility, audits must be performed by individuals who have completed standardized training provided by the Welfare Quality^®^ Network (WQ Network) [5].

Due to the complexity involved in assessing farm animal welfare, the time required for the application of the full WQP (around seven to eight hours on a typical farm with 200 dairy cows) has received significant criticism [5,8]. This raises concerns about the practicality of routinely using the full protocol by farmers [9]. Some studies have explored the possibility of accomplishing the assessment of a few individual measures or potential indicators that could reliably predict overall welfare classification, thereby reducing assessment time [8,9].

In early 2023, the Welfair^®^ scheme proposed simplified internal audits, focusing on just 10 indicators for assessing dairy cows on farms. Self-assessors can select five indicators based on their preferences, while the remaining five—body condition, water provision, lameness, integument alterations, and disbudding/dehorning—must be evaluated according to WQ Network guidelines. This approach simplifies the examination and shortens the assessment duration. However, these simplified audits provide only individual results for each of the ten indicators and do not yield a final classification score, which may limit their usefulness in ensuring farms are fully prepared for certification audits. No studies to date have investigated the feasibility of these simplified internal audits or their effectiveness in preparing farms for the certification audit.

Therefore, our study hypothesizes that the simplified internal audit assessment is crucial for enhancing welfare practices on farms, ultimately contributing to improved final classifications. Our aim is to determine whether simplified audits enhance welfare practices on farms and if they provide reliable risk assessments within the Welfair^®^ scheme. We believe that our work will provide valuable insights into the Welfare Quality^®^ Network and the implementation of simplified internal audits.

## 2. Materials and Methods

The present study was conducted on seven commercial dairy farms (main dairy cattle breed: Holstein Friesian) in continental Portugal from April to July 2023. Each farm underwent an initial assessment using the simplified WQP for dairy cattle, performed by a trained veterinarian (simplified internal audit). Following this, an assessor from a certification company performed the full WQP (official certification audit). To avoid bias assessment, none of the assessors were informed that the results of the visits would be analyzed in this study. All farms were in their first year of the Welfair^®^ Certification scheme.

Table 2 provides detailed information on the location of each farm, the size of the dairy herd (which ranges from 70 to 1260 cows, with an average of 320 cows per farm), and the interval, in days, between the two welfare assessments: the simplified audit and the certification audit (averaging 52 days). The simplified internal audits were performed always by the same assessor; Assessor 2 performed all certification audits except for Farms F and G (Table 2). Six of the seven farms included in this study are located in the northwest region, considered the main milk-producing region in Portugal.

### 2.1. Welfare Assessment

Sampling of lactating and dry cows was determined according to WQP guidelines [5]. On farms where not all animals were included in the study, cows were randomly selected and marked on their withers. Resources or management measures such as pain management in disbudding/dehorning, access to an outdoor loafing area or pasture, mortality rate, and dystocia cases were provided by the farmer. All assessors (the veterinarian with animal welfare consultancy experience for the simplified audit and the technicians from the certification company) were previously trained to apply the Welfare Quality^®^ assessment protocol for dairy cattle [5] in a three-day course recognized by the WQ^®^ Network and the Welfair^®^ scheme managers.

#### 2.1.1. Data Collection by the Simplified Internal Audit

Data collection was carried out, whenever possible, in the morning after milking to facilitate the assessment of clinical indicators during feeding time. When this was not feasible, the simplified internal audit was carried out at a time that best aligned with the farm’s routine. Ten indicators were measured at the farm: five of them were chosen by the assessor (avoidance distance, ocular discharge, body cleanliness, mortality rate, and milk somatic cell count), and the other five were the ones proposed by the Welfair^®^ scheme (body condition, water provision, lameness, integument alterations, and disbudding/dehorning). For the present study, only the 5 measures selected by the Welfair^®^ scheme were analyzed, since those measures must always be assessed, regardless of the dairy farm conditions or preferences. All measures were assessed according to the guidelines of the WQP for dairy cattle, Version 2, 2016 [5].

#### 2.1.2. Data Collection from the Certification Audit

Data collection was always carried out in the morning after milking or during the first feeding time of the day. A Portuguese certification company, aware that the farms had been previously evaluated by an animal welfare consultant, conducted the certification audits, with 2 different assessors performing these visits (Table 2). The on-farm visit involved a comprehensive application of the WQP for dairy cattle, which encompasses the four welfare principles, 12 criteria, and 30 measures (detailed in Table 1 of Section 1).

### 2.2. Scores

#### 2.2.1. Scores from the Simplified Internal Audit

Based on the data collected at the farm, each of the five indicator results was categorized as insufficient, sufficient, good, or excellent, as outlined in the WQP for dairy cattle [5] (Table 3). As mentioned before, there is not a final representative score of this audit, so whenever a measure was classified below good, a corrective action plan was developed by the animal welfare consultant, including recommendations tailored to the farm’s routine, economic factors, and housing features.

#### 2.2.2. Scores from the Certification Audit

The data recorded was further processed using the software program of the WQ scoring system (available online: https://www1.clermont.inra.fr/wq/wq_old/) (accessed on 1 September 2023), enabling us to finally classify each farm into 4 different welfare categories: not acceptable (<20 points), acceptable (20–54), enhanced (55–79), or excellent (80–100) [5]. As the final report also summarized the indicators’ results, it was possible to obtain the scores of the 5 indicators that are evaluated in the simplified audit.

More detailed information can be found in the WQP for dairy cattle [5].

### 2.3. Analysis of the Results

Insertion and organization of data were carried out using the program Microsoft^®^ Excel 2016. Data were divided accordingly between the two moments of assessment by farm. A descriptive analysis was carried out between the results of the common five indicators of each audit and the final classification of the certification audit.

## 3. Results

### 3.1. Individual-Level Indicators

**Figure 2:**
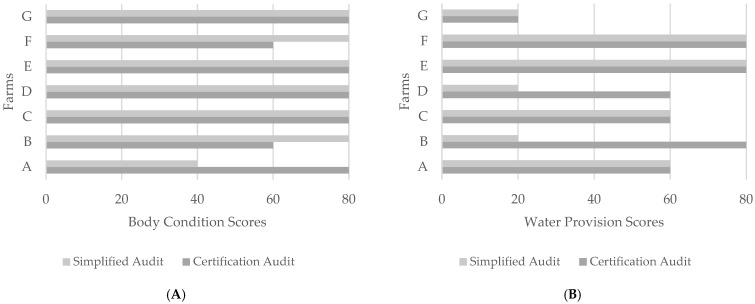

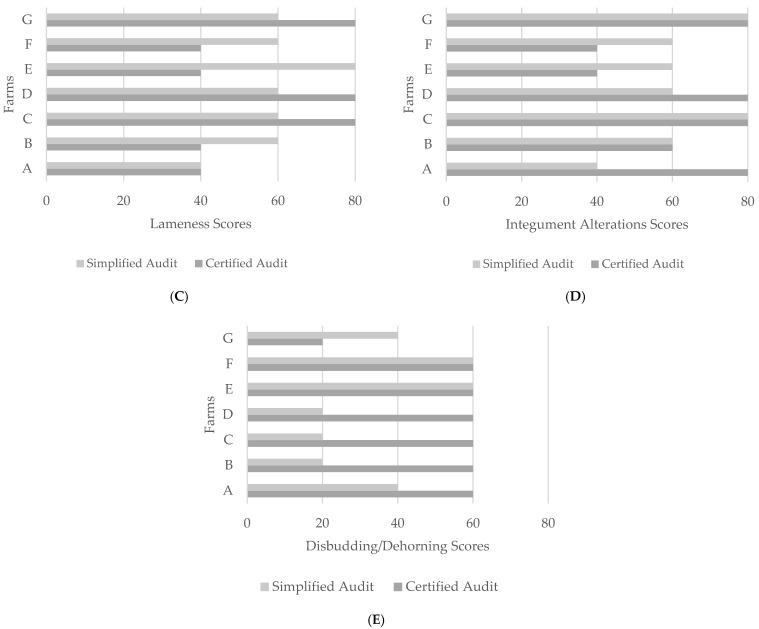
(**A**–**E**) Results of the five indicators assessed at both audits by farm (A–G): (**A**)—body condition; (**B**)—water provision; (**C**)—lameness; (**D**)—integument alterations; and (**E**)—disbudding/dehorning. To facilitate the interpretation of results, the x-axis of each graph represents the score result as follows: 20—insufficient; 40—sufficient; 60—good; and 80—excellent.

The results of the five indicators measured at both audits (body condition, water provision, lameness, integument alterations, and disbudding/dehorning) are shown in Figure 2A–E by the farm (A–G).

### 3.2. Final Classification Scores

Table 4 provides the farms’ final classification score resulting from the official certification audit. There is no farm’s final classification score resulting from the simplified intern audit.

## 4. Discussion

The ‘Body Condition’ indicator exhibited the most consistent assessment scores across visits. Notably, Farm A showed a significant improvement in body condition score following the simplified visit. In this case, it was recommended to collaborate with the farm’s nutritionist to review the quality of the diet, as well as to address questions regarding feed quantity and distribution routines. Nonetheless, body condition scores are inherently influenced by the cows’ reproductive phase, and these recommendations aim to address, but are not solely confined to, this variability. Identifying daily practices that could positively impact body condition scores on dairy cows is essential to help promote improvements in this area. For ‘Lameness’ scores, it is essential to consider the interval between audits, which averaged 52 days. Weather conditions and seasonal effects can influence clinical parameter assessments [10]. A study by [11] found that correlations for lameness varied from 0.48 to 0.78 during consecutive visits to dairy farms over five bimonthly intervals, highlighting how these factors may explain score discrepancies between audits. Even so, assessing lameness during the simplified audit provides valuable information, enabling farmers to establish a hoof-trimming schedule tailored to the specific needs of their farm. Recommendations for hoof-trimming frequency rely on several factors, such as housing conditions, management practices, and the farm’s history of lameness, which underscores the importance of including this parameter in the audit process.

Integument alterations are often indicative of the comfort of the resting area. For instance, cows housed in sand-bedded free stalls exhibit fewer hairless patches, wounds, and a lower incidence of lameness compared to those in facilities with other surfaces, such as rubber mats, mattresses, or straw [12,13,14]. The surface on which cows lie is crucial for their welfare, as they spend most of their day lying and ruminating [15]. Lesions such as swellings at the knee or hock joints can result from suboptimal bedding conditions or inadequate dimensions of the laying area, while skin alterations around the neck may stem from the feeding system designs. During the simplified visit, it is the assessor’s responsibility to identify these issues and recommend corrective actions to prevent severe integument alterations, which contribute to improved final scores. However, fluctuations in cleanliness and dryness of the lying material can also impact these animal-based measures between visits, which may explain occasional score disparities.

Analyzing the overall results of animal-based indicators (body condition, lameness, and integument alterations), no consistent improvement was observed across all farms, even after recommendations outlined in the corrective action plan. This raises concerns about the goal of the simplified internal audit, as these indicators may still vary during the year due to extrinsic factors (e.g., environmental conditions, daily farm routines) or cows’ intrinsic factors, and the simplified visit on its own cannot address all these variabilities and propose effective solutions. This opens the discussion of whether farms should be visited more than once a year by an animal welfare consultant to fulfill all aspects related to the farm’s welfare status.

Water provision, a resource-based measure, displayed the most significant score discrepancies between audits on Farms B and D. This is noteworthy, as the primary aim of simplified audits is to prepare farms for certification, promoting changes in resource-based measures critical for maintaining long-term welfare. While animal-based measures are generally preferred for assessing actual welfare status [16], a study by [10] showed that water provision measures were consistent over time in fattening cattle farms assessed at six-month intervals. In these specific cases, recommendations included adding water points to ensure each stall provided at least 6 cm of drinking space per animal or a minimum of one bowl for every 10 animals, along with at least two drinkers per stall. Enhanced cleaning routines for water troughs were also advised. Following the initial visit, both farms implemented these recommendations, resulting in the highest scores for this indicator on the certification audit. Farmer motivation to adopt improved practices is crucial to ensure these changes are effectively implemented.

Furthermore, research by [8] indicated that the “Absence of Prolonged Thirst” predicted overall welfare classification in 88% of cases, potentially reducing assessment time by up to 15 min. Although water trough design and flow rate are vital for the production and welfare of dairy cattle [8,17], concerns remain regarding the aggregation system of the WQP.

Farms A, B, C, and D achieved higher scores between audits due to changes in disbudding practices. Legally, farmers must ensure the welfare of the animals in their care, preventing unnecessary pain, suffering, or injury [18]. To ensure compliance, the method of disbudding is discussed during the first visit, stressing the importance of using hot-iron cauterization with anesthesia and analgesia in young calves. Most farmers still do not perform correctly due to limited willingness to pay the cost of medication, even though they recognized that disbudding causes prolonged postoperative pain (≥6 h) [19]. Moreover, caustic paste remains a common alternative [20], but recent studies have shown that calves perceive this method as a more negative experience than the use of hot-iron cauterization, even with sedation, local anesthesia, and analgesia [21]. This method is less recommended due to the high possibility of incorrect use and accidental burns in calves and operators [19]. The Portuguese authority (Direção Geral de Agricultura e Veterinária [DGAV]) has published technical guidance recommending hot-iron cauterization with anesthesia and postoperative analgesia for young calves [22], emphasizing the importance of evaluating this measure during simplified audits. The implementation of the simplified audit proved critical in guiding and correcting disbudding practices on farms after implementing the corrective action plan proposed by the animal welfare consultant. For instance, Farm A was initially not using anesthesia during disbudding; however, it was recommended, supported by the farm’s practitioner, to incorporate anesthesia in the disbudding protocol. The other farms, although utilizing hot-iron cauterization as their selected dehorning method, similarly lacked the use of analgesics and anesthesia before the simplified visit.

In Portugal, producers or non-veterinary technicians may administer local anesthetics or analgesics for dehorning, as long as they have undergone documented training held by veterinarians [22]. The simplified audit highlighted the importance of adhering to the technical guidance from DGAV, which facilitates practical implementation of improved disbudding practices on farms. None of the farms achieved the highest score, as this would require the complete elimination of calf disbudding or dehorning—a practice currently widely refused by dairy farmers.

In the overall assessment, the farmer’s motivation was found crucial for adopting recommended practices. Providing valuable guidance that aligns the farm’s conditions and management routines is essential. Additionally, understanding the farm’s economic situation and the farmer’s openness to progress is important to ensure successful implementation. The simplified visit, together with the recommendation action plan, proved useful for farmers in identifying procedures that needed improvement based on the assessed indicators. These implemented changes should be maintained, as the requirements of the full certification audit must be met annually. There is, of course, the danger that improvements are held provisionally (up to the official certification), so regular visits by a welfare consultant are advisable.

Poor interobserver reliability could justify the discrepancies seen between assessments, even though several studies [4,10,23] have already found good levels of interobserver agreement. Conversely, observers’ experience and background may also account for the discrepancies observed between assessments. This emphasizes the necessity of holding frequent training meetings to reduce the risk of low reliability when evaluating animal-based indicators, which are more prone to variation [4].

Similarly to what happened in the studies of [8,24], none of the farms assessed in our work were excellent, being limited to enhanced overall classifications. Farm G was the only one to fail the certification audit (with an overall classification of ‘acceptable’). The small interval time between the audits (9 days) made it impossible to address all the issues pointed out in the simplified audit. For instance, it restricted Farm G’s ability to make recommended changes to their disbudding procedures, which included the addition of analgesics in future interventions, as well as alterations regarding drinking points, obtaining the same score in this indicator as the simplified audit. Farms E and F, despite also having a restricted timeframe between visits, had already implemented the best disbudding procedures and better water provision scores, requiring no significant changes. This underlines the importance of giving time to make all the necessary changes.

Focusing exclusively on these five indicators may neglect other important indicators, as none of the five selected indicators belong to the ‘Good Housing’ and ‘Appropriate Behavior’ principles. This oversight undermines the principles as integral components of the WQP, as the visit should encompass all four principles.

Ideally, a direct comparison between the simplified audit and the certification audit scores should have been made in this study. However, this was not feasible as the simplified protocol does not yield an overall score, complicating accurate risk analysis. Although our results underscore the helpfulness of simplified internal audits in driving changes in farm routines, auditors cannot ensure that the farm is fully prepared for the certification audit by only relying on the simplified visit. The lack of a conclusive score becomes particularly problematic when a farm falls into a ‘gray area’—not performing exceptionally well, yet not poorly either. In such cases, without a comprehensive final score, it is challenging to determine whether the necessary improvements are met based solely on the individual indicator scores assessed.

## 5. Conclusions

Overall, the study’s results suggest that if farmers are motivated to improve their farm’s welfare status following the simplified internal audit, these visits can significantly contribute to animal well-being and foster improved welfare practices, which may positively influence final classifications. This can be further ensured by establishing regular visits by a welfare assessor.

However, this preliminary study raises awareness about the lack of consensus regarding simplified visits. Future efforts should aim for a more standardized assessment, as audits can be performed without including any indicators from the ‘Good Housing’ and ‘Appropriate Behavior’ principles. Additionally, developing a scoring model to facilitate internal audits and generalize a final score would be a valuable tool for auditors and farmers.

## Figures and Tables

**Table 1:** Welfare Quality Protocol for dairy cows on farms [5]. Resource-based measures are highlighted in bold, and management-based measures are underlined.

Welfare Principles	Welfare Criteria	Assessed Measures
Good Feeding	Absence of prolonged hunger	Body condition score
	Absence of prolonged thirst	**Water provision; cleanliness of water points; water flow; functioning of water points**
Good housing	Comfort around resting	Time needed to lie down; animals colliding with housing equipment during lying down; animals lying partly or completely outside the lying area; cleanliness of udders, flank/upper legs, lower legs
	Thermal comfort	As yet, no measure has been developed.
	Ease of movement	**Presence of tethering;** access to outdoor loafing area or pasture
Good health	Absence of injuries	Lameness; integument alterations
	Absence of disease	Coughing; nasal discharge; ocular discharge; hampered respiration; diarrhea; vulvar discharge; milk somatic cell count; mortality; dystocia; downer cows
	Absence of pain induced by management procedures	Disbudding/dehorning; tail docking
**Appropriate behavior**	Expression of social behaviors	Agonistic behaviors (head butts; displacements; chasing; fighting; chasing-up)
	Expression of other behaviors	Access to pasture
	Good human–animal relationship	Avoidance distance
	Positive emotional state	Qualitative behavior assessment

**Table 2:** Overview of the farms included in the study (A–G).

Farm	Location	Size of Dairy Herd	Interval Between the Two Welfare Assessments (days)	Assessor of the Simplified Audit	Assessor of the Certification Audit
A	Northwest Portugal	70	84	Assessor 1	Assessor 2
B	Northwest Portugal	95	84	Assessor 1	Assessor 2
C	Northwest Portugal	80	84	Assessor 1	Assessor 2
D	Northwest Portugal	210	85	Assessor 1	Assessor 2
E	Northwest Portugal	270	10	Assessor 1	Assessor 3
F	Northwest Portugal	1260	8	Assessor 1	Assessor 3
G	Central Portugal	200	9	Assessor 1	Assessor 2

**Table 3:** Score description for the five indicators proposed by the Welfair^®^ scheme (body condition, water provision, lameness, integument alterations, and disbudding/dehorning) [5].

Indicator	Score	Score Description
Body Condition	Excellent	When less than 2.68% of animals have poor body condition.
	Good	When less than 7.51% of the animals have poor body condition.
	Sufficient	When more than 7.52% and less than 33.38% of the animals have poor body condition.
	Insufficient	When ≥33.38% of the animals have poor body condition.
Water Provision	Excellent	When there are ≥6 cm of drinkers by animal or at least 1 bowl for each 10 animals, at least 2 drinkers by stall, and they are clean.
	Good	When there are ≥4<6 cm of drinkers by animal or at least 1 bowl for each 15 animals, at least 2 drinkers by stall, and they are clean. Or when there are ≥6 cm drinkers by animal or at least 1 bowl for each 10 animals, they are clean, but only 1 drinker by stall.
	Sufficient	When there are ≥4<6 cm of drinkers by animal or at least 1 bowl for each 15 animals, but drinkers are not clean.
	Insufficient	When there are <4 cm of drinkers per animal or less than 1 bowl per 15 animals.
Lameness	Excellent	Less than 2.5% of the animals are lame.
	Good	Less than 8.11% of the animals are lame.
	Sufficient	More than 8.12% and less than 29% of the animals are lame.
	Insufficient	More than 30% of the animals are moderately lame.
Integument Alterations	Excellent	Less than 8.75% of the animals have integument alterations.
	Good	Less than 21.26% of the animals have integument alterations.
	Sufficient	More than 21.27% and less than 49% of the animals have integument alterations.
	Insufficient	More than 50% of the animals have integument alterations.
Disbudding/Dehorning	Excellent	Less than 15% of the animals are disbudded/dehorned.
	Good	Performance of disbudding in more than 15% of the animals with iron cauterization or caustic paste with anesthesia and analgesia.
	Sufficient	Performance of disbudding in more than 15% of the animals with iron cauterization or caustic paste with anesthesia or analgesia.
	Insufficient	Performance of disbudding in more than 15% of the animals with iron cauterization or caustic paste without anesthesia and analgesia.

**Table 4:** Final classification scores of the seven farms (A–G).

Farm	Score	Final Classification
A	55	Enhanced
B	55	Enhanced
C	66	Enhanced
D	55	Enhanced
E	57	Enhanced
F	55	Enhanced
G	42	Acceptable

## Data Availability

The data supporting the reported results are available upon reasonable request to the corresponding author. The data are not publicly available due to privacy reasons.
